# Digital Transformation Led by Nurses and Nursing Managers' Priorities: A Qualitative Study

**DOI:** 10.1155/2024/8873127

**Published:** 2024-08-24

**Authors:** O. Navarro Martínez, J. M. Leyva-Moral

**Affiliations:** ^1^ University of Valencia Faculty of Nursing and Podiatry Nursing Department, Valencia 46010, Spain; ^2^ Autonomous University of Barcelona, Cerdanyola del Vallès, Barcelona 08193, Spain

## Abstract

Nurse-led digital transformation promotes nurses' participation and leadership in digital health. It also aims to improve the quality of care and patient satisfaction. The objective is to describe the priorities of nursing managers in the field of digital health and their beliefs related to the digital transformation led by nurses. The methodology used is a qualitative-descriptive study. The data were collected by means of the implementation of playful-reflective workshops. Open and/or closed questions were used, to which participants responded using their mobile phones, by capturing QR codes. A group of 32 nursing managers from the Spanish private hospital sector were invited to participate by means of purposive sampling. Data were analysed using the Braun and Clarke thematic analysis performed by two researchers independently. Of the 32 participants, 25 were women. The average age was 38 years. The main utility of the use of technology in health environments that they noted was innovation, followed by communication. Another utility mentioned repeatedly was that of visibility, considering digital media as a way to show the population the role of a nurse. They also noted time savings and error reduction. Moreover, as barriers to the implementation of technology, participants pointed out the lack of skills or equipment, institutional support, and the care burden. This study shows that nursing managers are aware of the need and benefits that nurse-led digital transformation can bring about. These findings can pave the way for promoting a nurse-led digital leadership culture.

## 1. Introduction

Digital health or e-health is not only a trend or a fashion but also a necessity that healthcare professionals must incorporate into their daily practice to establish new channels of communication with the patient and society, as well as to maintain lifelong learning and build knowledge with other professionals and institutions globally [1, 2]. Digital health is defined by the WHO as the cost-effective and safe use of information and communication technologies in support of health and related areas, including clinical care services, health surveillance, health literature and health education, knowledge, and research [3]. In this regard, public and private organizations implement e-health systems in health centres and among health professionals as a way to reach the patient, save costs, streamline procedures, etc. [4–6]. The benefits of digital health for patients have been promoted in numerous publications in recent years, either in the form of exchange communities [7–9] or at the individual user level [10–13]. Digital health literacy can be seen as a useful tool for health education, promoting healthy lifestyles, reducing complications, or monitoring patients with chronic diseases [14, 15]. This is especially relevant in times of uncertainty like what happened during the COVID-19 pandemic, where not only cost reduction or immediacy was crucial but also protecting patients by minimizing unnecessary visits to health centres was crucial [16, 17]. In this sense, authors such as Fronczek et al. consider that the arrival of the pandemic has nurtured nurses' use of the Internet and social networks as an amplifier of their voice, a way to reach communities easily and quickly, influence health policies, and exercise active leadership [18]. In fact, nurses are in a privileged place in the health system because they are the most numerous professionals in all health systems worldwide and have an important role in patient education and follow-up. Nurses, through their work, contribute to improving patients' ability to understand and use health information for their own health [19]. However, while the use of digital media implies a proactive and positive attitude, it also requires training; few healthcare systems are committed to the lifelong training in digital skills [20] although successful implementation requires, especially, institutional support [21]. The incorporation of technology in patient care is the responsibility of the system as a whole and cannot depend only on the goodwill or interest of professionals, and it must be supported by institutions and collegiate organizations, as well as regulated by the state at a higher level and included in the national curriculum [21, 22]. We have rephrased the sentence “This is especially relevant … health centres was crucial” for clarity. Please confirm that this is your intended meaning.

Nurse-led digital transformation (NLDT) is an approach that focuses on the participation and proactive leadership of nurses in all facets of digital health [23]. This transformation is a journey of improvements and changes that focus on patients, practice, and education. NLDT incorporates digital tools to improve the quality, safety, and integrity of nursing care. This includes the use of innovative technologies such as robotics, telemonitoring, and the use of mobile applications or devices to improve the quality of nursing care [24]. Therefore, this digital transformation aims to improve the quality of care and it increases patients and nurses' satisfaction [25].

In Spain, it is known that the lack of institutional support is an important barrier when applying technology in nurse practice [20]. Internationally, several authors also point to healthcare organizations as responsible for facilitating resources and spaces for the use of technology, as well as providing healthcare professionals with the time and opportunities to learn how to use them [21]. This is especially relevant for nurses who have not received training in this area during their undergraduate studies [22]. As our previous studies were based on nurses without management positions, we consider in this case that the institutions are led and managed by managers, and therefore, it is interesting to know their vision on this issue. However, the beliefs of nurse managers regarding digital transformation and the professional use of such technology are not well known yet. Therefore, the objective of this study is to describe the phenomenon of digital transformation led by nurses from the perspective of nursing managers. A detailed understanding of this new area of nursing will identify barriers, limitations, facilitators, and possible applications, helping to offer possible solutions to integrate technology in the field of care. This study is a starting point when focusing on identifying the weaknesses and strengths of NLDT. Only by being aware of these limitations will nurses be able to advance in professional development, including care in the digital world as part of new skills [26].

## 2. Materials and Methods

### 2.1. Study Design

This study used a qualitative-descriptive design. This type of study provides a detailed description of the bias aspects of events or experiences from a subjective perspective [27]. In addition, a qualitative-descriptive design allows capturing the richness and complexity of the phenomenon under study [28] and provides a complete and detailed description of a phenomenon without extensive interpretation or theorization [29]. The research followed the Consolidated Criteria for Reporting Qualitative Research (COREQ) [30].

### 2.2. Participants and Instruments

The study involved nursing managers from private hospitals in Spain. Their participation formed an integral component of a voluntary training initiative offered by their respective institutions. The present study undertook a qualitative examination of the data collected, specifically pertaining to the digital transformation spearheaded by nurses. The data were collected through the facilitation of playful-reflective workshops. These workshops were designed as collaborative workspaces, engineered to foster collective engagement and reflective inquiry within work-play-reflection sessions. Such sessions are meticulously structured, incorporating a diverse array of participatory tools [31], with group dynamics and techniques meticulously tailored to align with a central theme and objective [32]. Both open-ended and closed-ended inquiries constituted the framework for data elicitation, with participants leveraging their mobile devices to respond via a QR code embedded within the presentation materials. Further elaboration on the utilized tools and their respective functionalities is detailed in Table 1.

Table 2 shows the questions asked, as well as the tool used, and a brief explanation of the mode of question or activity used. The questions included in this study are of our own elaboration, based on the results obtained in previous studies. For the questions about competencies, the authors have followed the digital competency framework established by the Spanish Ministry of Education [33].

This type of tool has already been used in previous studies in qualitative research contexts for the collection of opinions and generation of subsequent debate among professionals [34–36] and also with patients [37].

For ideas and the generation of group work, Miro is a useful option to foster collaboration among students, giving them a workspace to generate ideas freely and share resources [38]. In addition, Miro boards encouraged creativity and promoted a collaborative, meaningful learning experience [39]. Each response was coded by assigning a number to each participant to make it easier to work with the data.

### 2.3. Data Collection

The health system in Spain is a system in which public hospitals (financed 100% by the State) and private hospitals (financed by private capital) coexist and even collaborate. These private hospitals serve patients who pay to receive benefits and patients referred from the public system who do not pay to receive assistance, due to agreements with the State. Private hospitals in Spain represent 56% of all hospitals. The data collection phase was overseen by the principal researcher, a female PhD holder, who assumed the role of instructor during the face-to-face course conducted between November 2022 and January 2023. These instructional sessions were held within the academic confines of a university situated in Madrid. Engaging in this initiative were nursing managers actively partaking in a voluntary lifelong learning endeavor facilitated by their respective private hospitals spanning Spain. Noteworthy is the diverse spectrum of professional experience exhibited by these nursing managers in their roles. The duration of the session extended to four hours of intensive face-to-face training. Participants were apprised at the outset of the training regarding the qualitative analysis awaiting their submitted exercises, integral to the current study. Full elucidation regarding the study's objectives and procedures was provided. Participants were accorded the autonomy to determine the inclusion of their exercises in the study, with no repercussions ensuing from their decision. It is worth noting that all participating nursing managers consented to and expressed satisfaction with the incorporation of their exercises as primary data for this qualitative inquiry.

Data collection ended once the data saturation was reached [40]. Data saturation in qualitative research refers to the point at which gathering additional data no longer yields new or insightful information or perspectives related to the research questions or objectives [41]. It signifies that the researcher has obtained a comprehensive understanding of the phenomenon under investigation, and further data collection would not contribute substantially to the richness or depth of the findings. In practical terms, reaching data saturation implies that recurring themes, patterns, or categories have emerged consistently across the collected data, and new data points are unlikely to introduce novel insights or perspectives [42]. In the context of this study, which involved the analysis of exercises created by participants in a leadership workshop designed for nursing managers, saturation manifested in a manner that differed from the conventional paradigm. Saturation was deemed to have been achieved when the exercises ceased to proffer novel insights, and the analytical process left no inquiry or reflection unaddressed.

Prior to the face-to-face sessions, detailed information on the study was provided to participants by e-mail. Those who agreed to participate received new written and oral information and were given a space to clear up any doubts before signing the informed consent. Participants did not receive financial compensation for their participation. No data were collected that could reveal the identity of the participants. The data were treated confidentially with access only to the investigation team. The study was conducted according to the ethical and legal rules of the Declaration of Helsinki and the Good Clinical Practice of the European Union. This project was approved by the Ethics Committee of the Catholic University of Valencia with the code UCV/2022-2023/001. None of the invited participants refused to participate or dropped out.

### 2.4. Data Analysis

The research team entered the data into a database for subsequent thematic analysis, following the method proposed by Braun and Clarke [43]. For a correct triangulation of the data, that is, to verify and compare the information obtained by collecting data in different methods and sections [44], the textual elements were reviewed, and their categorization was carried out, later, by a second researcher. In this way, data analysis expanded, deepened, and reduced the possibility of misunderstandings, thus clarifying the meaning and veracity of the information obtained in the testimonies. Finally, the resulting report was discussed with the research group and, through reflective thinking and critical reasoning, changes were made until a consensus was reached.

### 2.5. Rigour

To ensure the credibility, transferability, dependability, and confirmability of the study, Guba's criteria were meticulously applied throughout the research process. This involved a collaborative effort between two experts well-versed in e-health and qualitative methods. Each expert independently reviewed the assigned codes, themes, and interview quotations, with any discrepancies resolved through discussion. Additionally, to mitigate the risk of misinterpretation, a native editor/translator conducted backtranslation. Continuous discussion and reflective thinking among all researchers at every stage of the study contributed to its rigour, credibility, and overall trustworthiness.

The methodological strategies employed in this study aligned with established practices for data trustworthiness, encompassing an audit trail, bracketing, coding checks, categorization, continuous feedback, ongoing data interaction, participant confirmation, peer debriefing, structural corroboration, and referential adequacy [45, 46]. The iterative process of data translation involved persistent discussions within the research team to safeguard data integrity [47]. Upholding the authenticity of meaning during dissemination [48] was ensured through the rigorous review of source and target language codes, categories, and exemplar quotes by three external bilingual experts. Additionally, the study's credibility and consistency were maintained by providing a detailed description of the data collection process and documenting results with quotations from the transcripts. Furthermore, findings underwent scrutiny and verification through consensus meetings involving the research team and two experts specializing in qualitative research and gender identity issues.

## 3. Results

Thirty-two nursing managers from private hospitals participated, of whom 25 were women. The average age of the participants was 38 years old. The centres in which they work are located in different geographical areas of Spain: Andalusia, Valencia, Catalonia, Madrid, Canary Islands, Basque Country, and Galicia. Thematic analysis of the data identified four themes:

- Utility and concept of NLDT
- Essential and advanced digital skills
- Barriers to NLDT
- Immediate applicability of the NLDT

This is a group of participants who identify themselves as users with moderate-advanced skills in the use of social networks and in the search for digital information, with moderate-competence in digital security issues, which are self-described as beginners in the field of digital content creation.

### 3.1. Utility and Concept of NLDT

From the speeches of the participants, the digital transformation led by nurses could be defined as the set of digital tools that allow nurses to facilitate their daily work, especially in the area of communication and improvement of clinical records, which is beneficial for both patients and professionals. The main utility attributed to the use of technology in healthcare environments is innovation, mainly associated with communication. The technological tools are described as elements that allow a rapid and agile dissemination of information, thanks to which it is possible to unify criteria that allow the progress of the implementation of unified registries. In short, according to the participating nurse managers, the use of information technologies allows them to give visibility quickly and effectively to certain interventions that, indirectly, improve professional and patient safety and contribute to optimizing key resources such as time.

**Figure 1:**
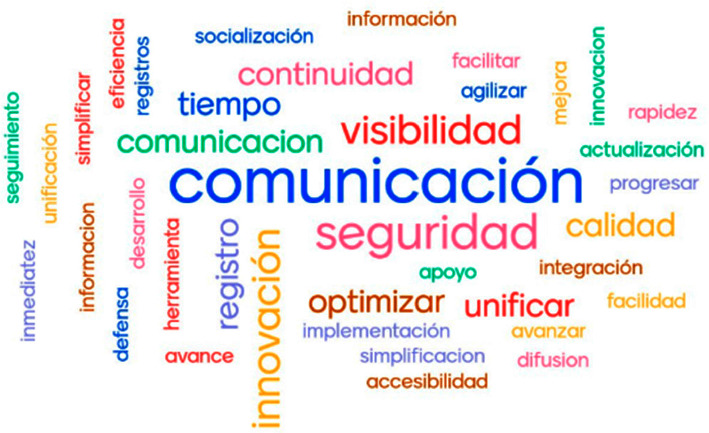
Word cloud with participants' contributions in question in short “what can technology be useful for?”

Figure 1 shows an example of a word cloud, in this case, related to the question of the usefulness of the technology.

*“I believe [NLDT] is the development and application of digital media in the world of nursing. It is that the staff of the world of nursing can create, develop and give visibility to our collective and serve us as a working tool.”* (Participant 3)

*“[NLDT] is the application of new technologies integrated into nursing work as a vehicle for improvement and innovation. It consists of adding digital tools to our work to improve results and communication.”* (Participant 11)

One of the benefits that also emerges recurrently in the responses of the participating nurses is the visibility of the profession, considering digital media as a way to show the population what the role of the nurse is and strengthen their professional identity.

*“[NLDT] is giving more vision to the profession through digital media.”* (Participant 7)

*“[NLDT] is to bring the nursing world to users through the digital world, publicising the work of the nurse.”* (Participant 8)

### 3.2. Essential and Advanced Digital Skills

In the workshops, participants were asked to agree on the essential digital competence for the professional performance of nurses. Communication is again identified as the first option, accompanied by collaborative actions. The group then decided that the next essential digital competence for nurses was information and digital literacy, and ultimately skills related to solving technological problems, Internet security, and digital content creation.

*“[NLDT] is a change in management by means of digital platforms or applications to facilitate access to everything, being nurses who guide or define users' needs.”* (Participant 2)

*“We need to have digital tools for the day to day of our profession and update the practice of nursing and new technologies.”* (Participant 9)

These results make sense when the group is asked to discuss which area they would like to have advanced skills. In this case, it highlights the need to improve the use of computer programs, create apps, solve technical incidents, and know how to create digital content.

*“[I would like] to have more knowledge in the field so that I can put it into practice and teach others.”* (Participant 13)

“[I would like] to create apps that I can use in my daily practice of my profession.” (Participant 22)

### 3.3. Barriers to NLDT

The barriers identified by the group for the regular use of digital technology for nurses were mostly ignorance or lack of the necessary tools and skills. The participants point out that they do not have a favourable infrastructure, which also refers to institutional culture and the feeling of belonging to the institution. In this respect, they indicate that an adaptive process is necessary to allow the incorporation of these new competencies but that the current reality of the system and the age of many professionals do not ease such adaptation. Similarly, a considerable number of participants indicated that the current care burden is not compatible with the acquisition of new skills such as ICT.

*“Having an idea, but not knowing how to develop it due to a lack of knowledge of technological management. In addition, there is a lack of computer devices in the workplace to enable their development.”* (Participant 17)

### 3.4. Immediate Applicability of NLDT

As participants engaged in lively brainstorming sessions, the rich tapestry of ideas surrounding the integration of digital technology within hospital environments began to unfold. A plethora of insights, totalling over 150, emerged, encapsulating the collective wisdom and foresight of nursing managers. Amidst the flurry of ideas, several overarching subthemes emerged, shedding light on the multifaceted potential of digital innovation in healthcare settings. From the efficient management of personnel to the seamless integration of newcomers, participants envisioned a future where digital solutions would revolutionize the administrative landscape of hospitals. In the realm of patient care, the concept of geolocation and waiting time optimization emerged as a central theme. Participants passionately discussed the possibility of utilizing digital tools to enhance patient tracking and streamline waiting times, thereby ushering in a new era of efficiency and patient-centred care.

*“I've been considering the advantages of a mobile app for tracking patients in the hospital. It could provide peace of mind to families by updating them on their loved one's location, whether in the ER, getting X-rays, or in surgery, fostering a deeper sense of connection and support.”* (Participant 15)

*“I've been reflecting on ways to improve patient admissions at our center. Imagine the convenience of bypassing long queues with a robotic admission system or similar technology. It has the potential to transform the patient experience, minimizing wait times and streamlining admissions for a more efficient process.”* (Participant 23)

The digital transformation of medical records emerged as another pivotal theme, with participants recognizing the transformative potential of digitalization in enhancing data accessibility and streamlining record-keeping processes. Beyond the confines of administrative and clinical realms, participants delved into the realm of patient empowerment and engagement. From self-management of nursing notes to personalized dietary management during hospitalization, the potential of digital technology to empower patients and enhance their healthcare experience was palpable.

*“I've been contemplating the advantages of a mobile app for hospitalized patients, offering guidance on nutrition, exercise, and medication. Such an app could truly empower patients to manage their health, enhancing their overall well-being during their hospital stay.”* (Participant 4)

*“We've discussed a proactive approach to prepare patients for appointments. Imagine receiving a helpful SMS just before, guiding you through each step, ensuring readiness and confidence for the upcoming consultation or procedure.”* (Participant 30)

As the discussions progressed, participants distilled their collective insights into three key areas of intervention deemed crucial for the future of hospital operations. The deployment of QR codes, applications, and wearable devices for patient geolocation garnered widespread support, with participants lauding its potential to enhance safety and traceability within hospital settings. Robotic assistance emerged as another focal point of discussion, with participants envisioning a future where robots would play an integral role in automating mechanical tasks, thus freeing up valuable human resources and enhancing operational efficiency. Finally, the importance of electronic onboarding support for new workers resonated strongly with participants, who recognized the pivotal role of digital technology in easing the transition process and fostering a culture of continuous learning and development within hospital environments. In essence, the collective brainstorming sessions offered a glimpse into a future where digital innovation would not only transform the operational landscape of hospitals but also empower patients and healthcare professionals alike, ushering in a new era of efficiency, safety, and patient-centred care.

*“Let's consider innovative solutions like mechanized robots for warehouse sterilization and pharmacy automation. These approaches promise to enhance efficiency and patient care, reflecting our commitment to exploring cutting-edge strategies for improvement.”* (Participant 26)

*“We're exploring ways to boost patient safety, particularly for new nurses. Consider a digital system that streamlines care processes—simplifying patient ID, medication tracking, and record-keeping—offering crucial support for novice nurses to deliver care confidently and accurately.”* (Participant 3)

## 4. Discussion

The nursing managers participating in this study present, in general, a positive and optimistic attitude regarding NLDT; in addition, they consider its implementation very useful and identified multiple utilities and benefits. These findings are in line with the Laukka study conducted with nursing managers in Finland [49]. The use of technological tools in the field of nursing care is not only a way to reduce errors and improve the effectiveness of interventions [21, 50, 51], but it also promotes the visibility and leadership of the profession. The results of this study coincide, therefore, with other authors who opt for the importance of digital competence and computer skills in the leadership of nurses [20, 49, 52, 53].

Another important finding of this research is the value of communication and collaboration in the digital field as a key competence for the development of nurses and the implementation of technology. Along the same lines, there are authors who point to collaboration as a driving force behind the adoption of digital tools [49–54]. It is evident, therefore, that communication and digital collaboration are competencies considered fundamental for nurses nowadays since they can allow them to advance in their professional and personal development. This could be due to the infinite possibilities offered by social networks and digital tools in a globalized and connected world to contact other professionals and learn from these exchanges. However, it is important to bear in mind that a significant digital division remains and that there may be significant differences due to generational, cultural, or even resource availability issues.

In this study, we observed how participants consider the use of technology as a priority for monitoring and follow-up, improving management, process performance, quality, and safety. These benefits coincide with those already obtained in other studies on this subject [55] and with publications that indicate that nurses need these skills to provide safe care to patients [22, 56]. It is clear that these benefits and concerns regarding the quality and safety of patients are a common point among management nurses, regardless of the context in which they work.

As shown in this research, nursing managers highlight barriers to the use of new technologies such as a lack of skills, as well as the high care burden and lack of institutional support. These reasons have already been contrasted in another previous research carried out by the authors in similar contexts [57]. Some authors also mention possible causes such as professionals not integrating ICT in their practice, a lack of time [58] as well as a lack of digital skills and competencies [59, 60]. The lack of institutional support and the low level of training that professionals receive make the gap between the demand of the population and the supply by health workers even more complex [21]. In the specific case of Spanish nurses, the lack of time is a real problem as it is far below the ratio of other countries according to the WHO, the OECD, and the General Council of Nursing [61].

It is interesting to note that, in this study, nursing managers consider the use of digital tools to accompany and train new nurses entering hospitals very useful. According to Sharpp [62], these tools would be an engine to generate motivation and even boost new leaders: leaders who promote relevant changes, bearing in mind that true leadership is someone capable of creating healthy work contexts, promoting self-efficacy, and motivating nursing leadership [63]. However, the state of digitalization in health varies considerably between European countries [64]. In Spain, only 44% of studies in nursing include training in digital competence in their curricula [1]. In other countries, such as Finland, public guidelines have been implemented in this area since 2015, helping to improve the level of digital competence of nurses [56]. Therefore, it would be advisable to have educational policies that integrate this competence transversely and continuously in the training of future nurses.

This research shows that nurse managers do not usually create digital contents and that they do not have competence in this area although they would like to have them as they consider them useful. Along the same lines, a quantitative report was published by the Signo Foundation in health managers in Spain [65]. This report also noted the participants' low content creation competence although in this case, only 12% were nursing managers. In this regard, Korte et al. also referred to the importance of nursing managers to generate engagement in the implementation of new technologies. In fact, this author points out that *Management actions can provide a structural framework and training so that nursing leaders can ensure their staff's engagement in using unknown devices, too* [53].

It is important to note that digital resources are powerful tools for the monitoring of people with chronic pathologies [66], and they can be used to understand the behaviour of patients in relation to health [67, 68] and be useful to educate the population and offer quality healthy advice [69]. Some authors, such as Rubel [70], insist on the need for professionals to lead the creation of quality content for patients, thus avoiding misinformation. However, the authors consider it essential that patients actively collaborate in this process of creating digital educational content, as other authors have [71].

This study presents a number of limitations that must be taken into account. The main limitation that can be cited in this study is the collection of data in groups. This data collection technique has advantages and disadvantages that should be assessed by the research team. In this case, we opted for group techniques given the active and creative dynamics of the activities, always looking for a summative discourse. With all participants holding similar positions, it was thought that there would be no hierarchical differences limiting speeches. The fact that all participants were managers in private institutions could also be indicated as a limitation. However, the fact of coming from different geographical locations within the country brings considerable richness to the data since the experiential discourse becomes summative given its heterogeneity. It is important to note that 78% of the participants in this study are women, which may not represent the reality of other institutions or geographical areas. According to WHO, only 25 per cent of senior positions in health organizations are held by women nurses [72].

## 5. Conclusions

This study shows how nursing managers value the NLDT positively. In fact, they consider digital tools to be useful for improving the safety and efficiency of nurses' work and, in addition, they underline their impact on improving the visibility and recognition of the profession at a social level. Along the same lines, managers consider that the most important competencies for the nursing profession are communication and collaboration. Despite the managers' identification of all these positive factors, they also mentioned factors that hinder digital leadership, such as the lack of digital competencies, the high burden of care, and the lack of institutional support. It is expected that the results obtained in this analysis will be useful to establish a basis for future studies, which could include mixed methodologies with a multicentre approach to identify and understand the barriers and facilitators faced by nurse managers when implementing technology in clinical services. This could be the starting point for educational, institutional, and monitoring proposals for technology integration to promote digital transformation and nurse leadership in this area. The nursing manager must be sensitive to NLDT, focusing on care, also in the digital environment, on professional-patient collaboration, giving the latter a leading role. Therefore, we recommend the use of participatory action research and other participatory qualitative methods by managers and nurses when designing materials, resources, and campaigns in the digital and technological environment.

## Figures and Tables

**Table 1:** Tools used and functionality.

Digital tool	Justification and usefulness
Slido https://www.slido.com/	This tool allows you to make questionnaires with different types of questions that can be answered interactively from a mobile phone. In this case, it was used to collect open answers. The answers are obtained anonymously and are displayed on the classroom screen, which allows interaction and discussion later during the session

Mentimeter https://www.mentimeter.com/	This tool also allows interactive questionnaires that attendees can answer from their phone. The questions used in this case were the word clouds (to start a topic or extract information about what most interests the group), test questions, and the visual scale (to perform group comparisons)

Miro https://miro.com/	This tool allows working in groups visually using brainstorming techniques, design thinking, canvas model, etc. In this case, brainstorming was used for collecting group ideas and later the bull eye diagram for prioritizing ideas

**Table 2:** Questions and modality used.

Question	Tool and type of question
*Applicability of technology in nursing*	
In short, what can technology be useful for?	Mentimeter
	Word cloud
What is nurse-led digital transformation for you?	Slido
	Free written reply
What possible uses could we give to technology in your hospital/service?	Miro
	Group brainstorming and brainwriting
Which of these ideas would you carry out first because you consider it a priority?	Miro
	Bull eye diagram for prioritizing ideas in a group

*Digital skills of nursing managers*	
I would say that I manage (on a scale of 5 points, being the worse mark—1 and the best mark—5) in:	Mentimeter Likert scale
(i) Social networks	
(ii) Searching for quality information	
(iii) In content creation: videos, blogs	
(iv) Safety issues in the network	
Which of the digital competences do you consider most useful for nurses' functions?	Mentimeter
	Sort through

*Constraints, barriers, and future expectations*	
If you could have super digital power, what would it be?	Mentimeter
	Short answer
Are nurses prepared to care in technology? (yes/no/I don't know)	Mentimeter
	Test type question
What barriers or limitations do we have to apply technology as a collective/individual level?	Mentimeter
	Short answer

## Data Availability

The data that support the findings of this study are available from the corresponding author upon reasonable request.
